# Data from proteomic characterization and comparison of mammalian milk fat globule proteomes by iTRAQ analysis

**DOI:** 10.1016/j.dib.2014.12.004

**Published:** 2015-01-13

**Authors:** Yongxin Yang, Nan Zheng, Xiaowei Zhao, Yangdong Zhang, Rongwei Han, Lu Ma, Shengguo Zhao, Songli Li, Tongjun Guo, Jiaqi Wang

**Affiliations:** a Ministry of Agriculture-Milk Risk Assessment Laboratory, Institute of Animal Sciences, Chinese Academy of Agricultural Sciences, Beijing 100193, China; b Institute of Animal Science and Veterinary Medicine, Anhui Academy of Agricultural Sciences, Hefei 230031, China; c State Key Laboratory of Animal Nutrition, Institute of Animal Science, Chinese Academy of Agricultural Sciences, Beijing 100193, China; d College of Food Science and Engineering, Qingdao Agricultural University, Qingdao 266109, China

## Abstract

Milk fat globules memebrane (MFGM)-enriched proteomes from Holstein, Jersey, yak, buffalo, goat, camel, horse, and human were extracted and identified by an iTRAQ quantification proteomic approach. Proteomes data were analyzed by bioinformatic and multivariate statistical analysis and used to present the characteristic traits of the MFGM proteins among the studied mammals. The data of this study are also related to the research article “Proteomic characterization and comparison of mammalian milk fat globule proteomes by iTRAQ analysis” in the Journal of Proteomics [1].

**Specifications table**

**Table:** 

Subject area	*Biology*

More specific subject area	*Milk proteomics*
Type of data	*Table, excel file*
How data was acquired	*iTRAQ labeling (Applied Biosystems)*
	*SCX chromatography and AKTA Purifier system (GE Healthcare)*
	*EASY-nLC 1000 system coupled with Q-Exactive mass spectrometery (Thermo FisherScientific)*
	*Proteome Discoverer 1.4 and MASCOT search engine (Matrix Science, London, UK; version 2.2)*
	*uniprot database (86 803 bovidae, 20 368 camelus, 28 583 horse, and 136 615 human entries, released in May 2014)*
Data format	*Analyzed*
Experimental factors	*Milk samples from Chinese Holstein cows, Jersey cattle, goats, Bactrian camels, horses, yak, buffalo and human were collected and used to systematically characterize the protein components of milk fat globules memebrane-enriched fractions.*
Experimental features	*Isobaric tags for relative and absolute quantification (iTRAQ)*
Data source location	*Beijing, China*
Data accessibility	*Analyzed datasets are directly provided with this article.*

**Value of the data**

• The data provide insight into the protein composition of milk fat globules memebrane-enriched fractions in parallel from the studied mammals.
• The bioinformatics data provide the potential physiological functions of the identified proteins.
• The data of multivariate analysis highlight the significance differences in the milk fat globules memebrane-enriched fractions among mammalian species.
• The data point out the breed-markers for identification of species specific milk fat globules.

## 1 Data, experimental design, materials and methods

### 1.1 Sample preparation

Milk samples were collected from 60 Chinese Holstein cows (*Bos taurus*) on a farm in Beijing, 21 Jersey cattles (*Bos taurus*) on a farm in Hebei, 27 goats (*Capra hircus*) on a farm in Shanxi, 21 Bactrian camels (*Camelus bactrianus*) and 18 horses (*Equus ferus caballus*) on a farm in Xinjiang, 24 yaks (*Bos grunniens*) on a farm in Qinghai, and 21 buffalo (*Bubalus bubalis*) on a farm in Yunnan. Ten human (*Homo sapiens*) milk samples were donated by healthy mothers between 3 and 8 months lactation and pooled.

The raw milk from each species was randomly pooled into three groups. Each group was treated as follows. Whole milk was centrifuged at 3000*g* at 4 °C for 15 min to recover the fat layer. The fat layer was incubated with PBS for 20 min at 37 °C and then centrifuged at 3000*g* for 30 min to obtain the floating fat layer. This procedure was repeated three times to recover the fat globules and remove residual caseins and whey proteins.

To analyze the fat globules, they were incubated with a lysis buffer containing 50 mM Tris–HCl, pH 7.4, and 4% (w/v) SDS for 1 h with periodic vortexing. Samples were then incubated in water for 5 min at 95 °C and centrifuged at 12 000*g* for 15 min. The floating cream layer was removed, the lysates were centrifuged again, and the supernatant was collected. Then, 200- μL aliquots of the protein mixtures were mixed with 1 mL acetone and stored at −20 °C for 20 h. Samples were then centrifuged at 14 000*g* for 40 min. Sediments were dissolved in lysis buffer and protein concentrations were determined by a modified Bradford assay (Bio-Rad, USA). Samples were stored at −80 °C.

### 1.2 Protein digestion

Two hundred micrograms of the protein mixtures were reduced with dithiothreitol at a final concentration of 100 mM, then incubated in 95 °C water for 5 min. After the samples cooled to room temperature, the sample was mixed with 200 μL UT buffer (8 M urea and 150 mM Tris–HCl, pH 8.0), loaded onto an ultrafiltration filter (10-kDa cutoff, Sartorius, Germany), centrifuged at 14 000*g* for 15 min, and washed again with UT buffer. Subsequently, 100 μL of iodoacetamide solution (50 mM iodoacetamide in UT buffer) was added to the filter, incubated for 30 min at room temperature in the dark, and centrifuged at 14 000*g* for 10 min. The samples were washed twice with 100 μL UT buffer. Finally, 100 μL dissolution buffer (Applied Biosystems, USA) was added and centrifuged at 14 000*g* for 10 min. This step was repeated twice, then 40 μL trypsin (Promega, USA) buffer (5 μg trypsin in 40 μL dissolution buffer) was added, and the sample was digested at 37 °C for 16–18 h. The filter unit was transferred to a new tube and centrifuged at 14 000*g* for 10 min. The digested peptides were collected and the peptide concentration was measured on a Nanodrop 2000 spectrophotometer at 280 nm [2].

### 1.3 iTRAQ labeling

A total of 30-μg peptide mixture was labeled with iTRAQ reagents according to manufacturer instructions (Applied Biosystems, USA). Human-derived samples were labeled with reagent 113, horse with reagent 114, goat with reagent 115, Jersey with reagent 116, Holstein with reagent 117, buffalo with reagent 118, yak milk with reagent 119, and camel with reagent 121. The labeling reaction was performed by 1 h incubation at room temperature.

### 1.4 Strong cationic-exchange chromatography separation

The labeled samples were acidified with 1% trifluoroacetic acid and separated by strong cationic-exchange chromatography with a Polysulfoethyl^TM^ (PolyLCInc, Maryland, USA) column (4.6×100 mm^2^, 5 µm, 200 Å) and an AKTA Purifier 100 (GE Healthcare, Maryland, USA). Buffer A consisted of 10 mM KH_2_PO_4_ (pH 3.0) and 25% (v/v) acetonitrile; buffer B was buffer A with 500 mM KCl. The column was equilibrated with buffer A for 32 min, and the samples were separated in 0–10% (v/v) buffer B for 10 min, 10–20% (v/v) buffer B for 5 min, 20–45% (v/v) buffer B for 5 min, 45–100% (v/v) buffer B for 5 min, and 100% (v/v) buffer B for 8 min at a flow rate of 1 mL/min. Thirty samples were collected and pooled into 10 fractions and then desalted on a C18 solid phase extraction column.

### 1.5 Liquid chromatography–tandem mass spectrometry analysis

Sample fractions obtained by cation-exchange chromatography were further separated and identified on a Thermo Fisher EASY-nLC 1000 system coupled with a Q-Exactive mass spectrometer. Buffer C consisted of 0.1% (v/v) formic acid in MilliQ water; buffer D was buffer C with 84% (v/v) acetonitrile. After the column equilibrated with 95% (v/v) buffer A, samples were loaded by an autosampler onto the trap column (2 cm×100 μm, 5 μm) and separated on the reverse-phase column (100 mm×75 μm, 3 μm) with buffer D in a segmented gradient at 250 nL/min as follows: 0–35% (v/v) buffer B for 100 min, 35–100% (v/v) buffer B for 8 min, and 100% (v/v) buffer B for 12 min.

Peptide analysis was performed on a Q-Exactive mass spectrometer in positive ion mode for 120 min, with a selected mass range of 300–1800 mass/charge (*m/z*). For the survey scan, resolving power was set to 70 000 at *m*/*z* 200, maximum ion injection time 10 ms, dynamic exclusion of the selected precursor ions was set to 40 s, and the automatic gain control target value was 3E6. MS/MS data were acquired using the top 10 most abundant precursor ions with charge ≥2, as determined by the survey scan. These were selected with an isolation window of 2 *m/z* and fragmented via higher energy collisional dissociation with normalized collision energies of 30 eV. For the MS/MS scans, resolving power was set to 17 500 at *m*/*z* 200, maximum ion injection time at 60 ms, and a 0.1% underfill ratio.

### 1.6 Protein identification and quantification

Raw files were processed in Proteome Discoverer 1.4 and then used to probe the MASCOT search engine (version 2.2; Matrix Science) of the selected species database. The database is an in-house uniprot database of bovidae, camelus, horse, and human with 86 803, 20 368, 28 583, and 136 615 entries, respectively (05-2014). The following parameters were applied: monoisotopic mass, trypsin as the enzyme and allowing up to two missed cleavages, MS/MS ion search, fragment mass tolerance at 0.1 Da, and peptide mass tolerance at ±20 ppm. Carbamidomethylation of cysteine, iTRAQ 8-plex (N-term), and iTRAQ 8-plex (K) were defined as fixed modifications, oxidation of methionine and iTRAQ 8-plex (Y) was specified as variable modifications. The decoy database pattern was set as the reverse of the target database. All reported data were based on 99% confidence for protein and peptide identification as determined by a false discovery rate (FDR) of no more than 1%, using 2 ⁎ N(decoy)/((N(decoy)+N(target)) to compute the FDR, in which the decoy is the reversed database and the target is the target database [3].

Relative quantification of identified proteins was performed in Proteome Discoverer 1.4 software. For peak integration, integration window tolerance was set to 20 ppm, integration method was set to most confident centroid. Relative peak intensities of released iTRAQ reporter ions were used to calculate the relative ratios of identified peptides to labeled samples (see Supplementary table 2). Relative quantification of identified proteins samples was calculated according to the weighted ratios of uniquely identified peptides that belonged to the specific individual protein (see Supplementary table 1). Final ratios were then normalized by the median average protein quantification ratio for all eight labeled samples that served as sample REF. This correction is based on the assumption that the expression of most proteins does not change. Protein identification inferred from the unique peptide identification in all experiments was considered. Differences in quantified proteins were analyzed by one-way ANOVA; differences between sample groups were evaluated by the Tukey׳s test; and *P*-values of less than 0.05 were defined as significant.

### 1.7 Bioinformatic and multivariate analysis

Analyses of the identified MFGM-enriched proteins associated with annotated functions were performed by the gene ontology (GO) annotation software (http://david.abcc.ncifcrf.gov/home.jsp) (see Supplementary table 3). Quantified proteins were processed by a principle component analysis (PCA) program in Unscrambler software (Camo, version 9.8, Norway). Cluster 3.0 software was used to investigate the hierarchical clustering of the identified proteins based on the logarithm of the intensities after data filtration. Java TreeView was used for data visualization (see table 2 and Figs. 1–3 in Ref [1]).
